# The Effect of Electroacupuncture on Microcirculation in Patients With Hypertension and Cognitive Impairment: Protocol for a Multicenter Randomized Controlled Trial

**DOI:** 10.2196/70876

**Published:** 2025-10-21

**Authors:** Jian Wen, XInlei Dong, Xiaolin Chen, Xiao Luo, Yuting Wang, Yimeng Gong, Kaixuan Ma, Dongling Zhong, Qinfeng Yan, Juan Li, Lili Zhang

**Affiliations:** 1 National Clinical Research Center for Chinese Medicine Acupuncture and Moxibustion First Teaching Hospital of Tianjin University of Traditional Chinese Medicine Tianjin China; 2 College of Acupuncture and Tuina Chengdu University of Traditional Chinese Medicine Chengdu China; 3 College of Health Preservation and Rehabilitation Chengdu University of Traditional Chinese Medicine Chengdu China

**Keywords:** acupuncture, mild cognitive impairment, hypertension, microcirculation, randomized controlled trial

## Abstract

**Background:**

Hypertension is a significant risk factor for cardiovascular diseases and is associated with an increased risk of mild cognitive impairment (MCI). The lack of effective treatment for these conditions underscores the urgent need for novel therapeutic approaches. Previous studies have indicated that microcirculation serves as the pathological basis for the comorbidity of hypertension and cognitive dysfunction. Our initial clinical studies have indicated that acupuncture could be a safe and effective treatment for managing hypertension and MCI. Whether acupuncture can enhance hypertension and cognitive impairment by modulating microcirculation, and the precise mechanisms involved, warrants further exploration.

**Objective:**

The objective of the trial is to evaluate the clinical efficacy of electroacupuncture on MCI of patients with hypertension and to explore whether it can improve hypertension and cognitive impairment by regulating microcirculation.

**Methods:**

In this multicenter, large-scale, single-blind, randomized controlled trial, we will recruit 252 patients with hypertension and cognitive impairment from 3 hospitals and randomly assign them to 3 groups in a 1:1:1 ratio: the electroacupuncture group, the sham electroacupuncture (SEA) group, and the waiting list group. The electroacupuncture group and SEA group will receive either electroacupuncture or SEA for 12 weeks, while the waiting list group will not receive acupuncture treatment for the first 12 weeks. The primary outcome will be the changes in overall cognitive function, as measured by the Montreal Cognitive Assessment. The secondary outcomes include blood pressure status, subdomain cognitive function, mental status, sleep quality, hemodynamics, and microcirculation indicators.

**Results:**

The study protocol has been approved by the institutional review board of the First Affiliated Hospital of Tianjin University of traditional Chinese medicine. This study was registered on April 26, 2024, with the Chinese Clinical Trial Registry. Data collection began in May 2024 and ended in April 2025. Currently, data from this trial are in the collection phase, and no data analysis has been performed. As of January 1, 2025, we have collected data from 65 patients. The results of this trial are expected to be submitted for publication in July 2026.

**Conclusions:**

This clinical trial aims to compare the efficacy of electroacupuncture versus SEA or waiting list control in the treatment of hypertension with cognitive impairment and to explore its impact on microcirculation through hemodynamic and microcirculatory indices. The results of this trial will contribute to clarifying the microcirculatory mechanisms of electroacupuncture in the treatment of hypertension with cognitive impairment, providing a solid foundation for further research on electroacupuncture therapy.

**Trial Registration:**

Chinese Clinical Trial Registry ChiCTR2400083501; https://www.chictr.org.cn/showproj.html?proj=220722

**International Registered Report Identifier (IRRID):**

DERR1-10.2196/70876

## Introduction

### Background

Mild cognitive impairment (MCI) represents a transitional stage between normal cognitive function and dementia. In China alone, the incidence rate of MCI ranges from 9.7% to 23.3% [1], and the annual conversion rate of MCI to dementia is estimated to be 5% to 20% [2]. Hypertension is an independent and significant contributor to cognitive impairment; it increases the risk of MCI by 1.62 times. From 2007 to 2011, the prevalence of hypertension with MCI (HTMCI) was 16.5% [3]; meanwhile, a meta-analysis published in 2023 showed that the overall prevalence of HTMCI in China was 37.6% [4]. Evidence suggests that hypertension can increase the risk of cognitive-related diseases, such as Alzheimer disease (AD) and vascular dementia [5]. A meta-analysis [4] published in 2021 revealed that the combination of high blood pressure and MCI is associated with a global prevalence of up to 30%. A *Lancet* study reported that over the past 30 years, the number of adults aged between 30 and 79 years with high blood pressure has risen from 650 million to 1.28 billion [6]. As the prevalence of hypertension continues to rise, the number of patients experiencing both hypertension and cognitive impairment is expected to increase.

There are established pathways from hypertension to cognitive impairment, which include atherosclerosis and arteriolar sclerosis [7]. Cerebral hypoperfusion and reduced neurovascular coupling can result in brain dysfunction by depriving energy-demanding areas of oxygen and glucose, essential for cognitive processes [8]. Hypertensive amyloid production and deposition might also contribute by promoting the pathogenesis of cognitive impairment [9]. Hypertension is widely recognized as an important risk factor for cognitive impairment. However, cognitive impairment can lead to limitations in daily behavior and activities [10], subsequently affecting the drug management and nursing routines of patients with hypertension, resulting in a vicious cycle [11]. Therefore, determining ways to prevent or delay the progression of hypertension accompanied by MCI represents an urgent clinical challenge.

Microcirculation is the comorbidity pathologic basis of hypertension and cognitive dysfunction. Microcirculatory dysfunction is closely related to the occurrence and development of cognitive impairment. Studies have shown that microvascular endothelial cell abnormalities and cerebral blood flow regulation disorders can lead to local tissue hypoxia, changes in blood-brain barrier permeability, and decreased metabolic waste removal efficiency, which in turn accelerates β-amyloid deposition and tau protein hyperphosphorylation through mechanisms such as neurovascular unit disorders, enhanced oxidative stress, and neuroinflammatory activation, eventually leading to neuronal damage and decreased synaptic plasticity. So, improving microcirculation (microcirculation disturbance [MD]) is a key link in the process of prevention and cure of HTMCI, mainly reflected in the occurrence and development of hypertension [12,13]. MD-based cerebral blood flow regulation disorder and cerebral hypoperfusion are closely related to cognitive function impairment [13]; MD is the common pathophysiological basis of hypertension and cognitive impairment: they interact and influence each other through microvascular thinning degree, hemodynamics, oxidative stress level, endothelial cell function, etc [14-16].

In recent years, numerous studies have demonstrated that acupuncture exhibits significant antihypertensive effects on mild to moderate essential hypertension [17-20] and is increasingly recognized as beneficial in improving cognitive function [21-24]. However, reports on the effect of acupuncture on patients with both hypertension and cognitive impairment are lacking. Our team conducted a randomized controlled clinical trial of large-sample, multicenter acupuncture treatment of essential hypertension in the early stage and found that after 6 weeks of electroacupuncture treatment, the 24-hour average systolic blood pressure in the treatment group was significantly decreased (mean 7.2, SD 11.0 mm Hg) compared with the sham acupuncture group and the waiting treatment group, and the antihypertensive effect lasted for 6 weeks [25]. Another experiment to observe the effect of acupuncture on cognitive function in patients with poststroke cognitive impairment found that acupuncture significantly reduced the overall cognitive function of patients with poststroke cognitive impairment compared with the sham acupuncture group and the waiting treatment group and had a certain lasting effect and also reduced the incidence of dementia [26].

### Objectives

On the basis of the theory of MD and our previous research, this study will conduct randomized controlled experiments to explore the effect of electroacupuncture on improving cerebral microcirculation in improving cognitive function.

## Methods

### Trial Design

**Figure 1:**
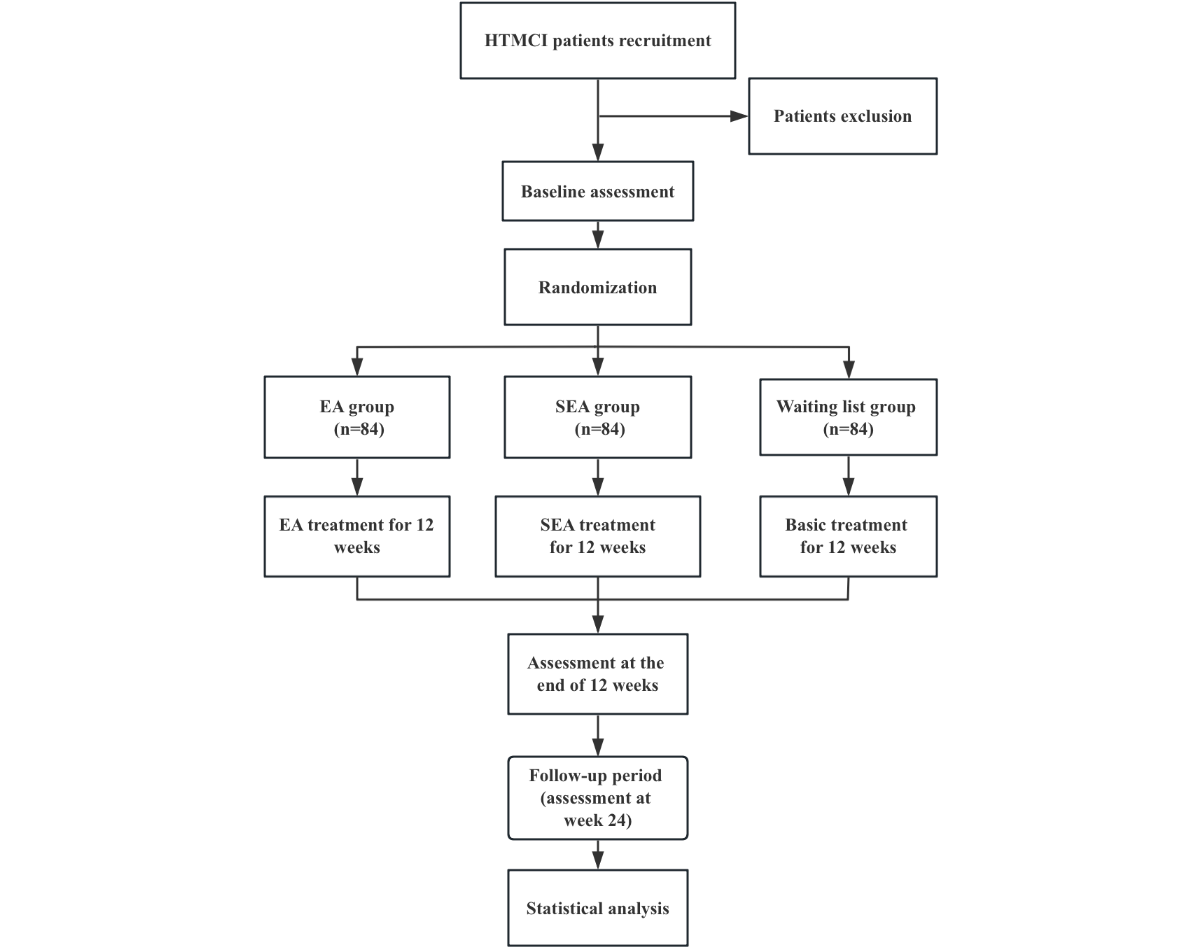
Flowchart. HTMCI: hypertension with mild cognitive impairment; SEA: sham electroacupuncture.

**Figure 2:**
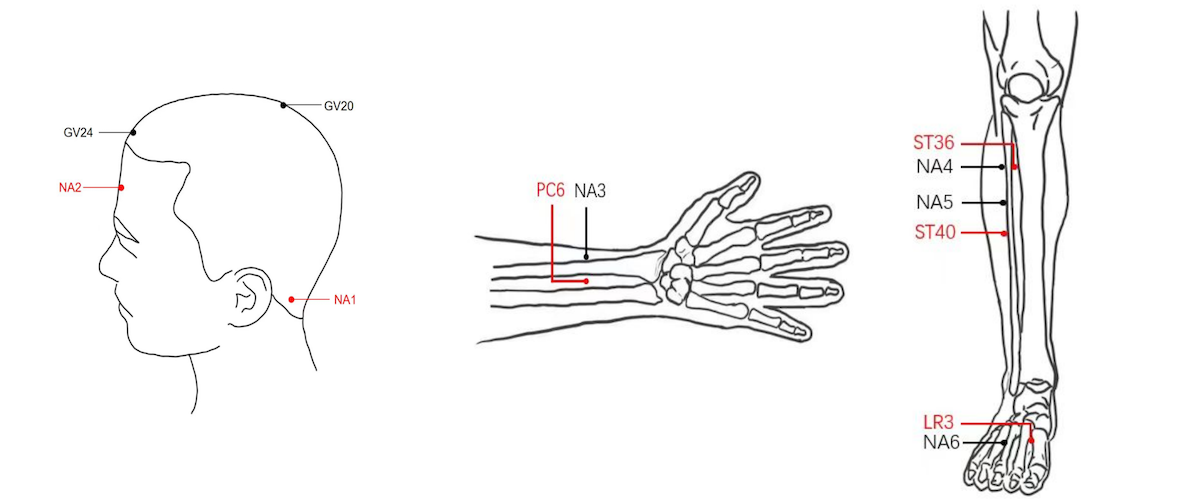
Location of acupoints and nonacupoints. Baihui: GV20; Fenglong: ST40; NA: nonacupoint; Neiguan: PC6; Shenting: GV24; Taichong: LR3; Zusanli: ST36.

This is a multicenter, open-label, waiting list control randomized controlled trial. A total of 252 eligible participants will be randomly assigned in a 1:1:1 ratio to the electroacupuncture group, the sham electroacupuncture (SEA) group, and the waiting list group. The assessments of clinical outcomes will be performed at baseline, week 12, and week 24. Acupuncture treatment was administered once every other day, 3 times per week, for 12 weeks, followed by a 12-week follow-up. The flowchart and study design schedule are shown in Figures 1 and 2, respectively.

### Ethical Considerations

The protocol has been approved by the institutional review board of the First Affiliated Hospital of Tianjin University of traditional Chinese medicine (TCM; TYLL2023{6}051). All patients provided their written informed consent to participate in the study and authorized the publication of their data. This study was registered on April 26, 2024, with the Chinese Clinical Trial Registry (ChiCTR2400083501). Every participant will be informed of detailed information about the study before signing informed consent. The results of this trial will be published in a peer-reviewed journal. Patients and the public were not involved in the design, or conduct, or reporting, or dissemination plans of this research.

### Participants and Recruitment

We will recruit participants in the outpatient departments of 3 hospitals, including the First Teaching Hospital of Tianjin University of TCM, the TCM hospital of Sichuan Province and Hospital of Chengdu University of TCM, the Affiliated Sichuan Provincial Rehabilitation Hospital of Chengdu University of TCM, and Tianjin Medical University General Hospital. Patients with HTMCI and healthy participants who are interested and whose initial screening meets our recruitment needs will be fully informed of the entire study procedure, benefits, and risks. Clinical trial communicators will fully explain the trial to patients before the trial begins to ensure that all the patients participate voluntarily and sign an informed consent form.

### Inclusion and Exclusion Criteria

The inclusion and exclusion criteria are presented in Textbox 1 [27-29].

**Inclusion and exclusion criteria.**

**Inclusion criteria**

- Meet the diagnostic criteria of primary hypertension [27]
- Meet the diagnostic criteria of mild cognitive impairment [28]
  Mini-Mental State Examination ≥21 points and <27 points
  Montreal Cognitive Assessment Scale ≥18 points and <26 points
  Activity of daily living scale <22 points, and no more than 1 item in the single score may be ≥3 points
- Aged between 40 and 75 years
- Primary school education (6 years) or above and able to correctly understand and complete the scale
- No acupuncture treatment has been received within 3 months
- Voluntarily cooperate and sign informed consent

**Exclusion criteria**

- Undergone treatments potentially interfering with cognitive functions, such as the use of cognition-impacting medications (eg, donepezil, memantine, and rivastigmine) [29]
- With neurological or chronic conditions, including systemic diseases (eg, anemia, tumors, and metabolic disorders) that could impair cognitive functions
- Presence of infection or focal injury, as indicated by brain magnetic resonance imaging, multiple infarcts, or cerebral white matter lesions in critical memory areas (Fazekas Rating >3)
- With secondary hypertension, refractory hypertension, or hypertension accompanied by clinical syndromes and target organ damage
- With aphasia, hearing impairment, muscle strength functional impairment below grade 3, severe visual impairment, or myopia exceeding 300, rendering them unable to cooperate with the test
- With coexisting diabetes, hyperlipidemia, or a BMI <18.5 or ≥28
- Who is considered by the researcher to be unsuitable for this study

### Randomization and Blinding

A total of 252 eligible patients will be randomly allocated to the acupuncture group, the sham acupuncture group, and the waiting list group at a 1:1:1 ratio. An independent professional statistician, uninvolved in the trial’s implementation or subsequent statistical analysis, will generate the randomization sequence using SPSS (IBM Corp) software. Both the outcome assessor and the statistician will remain blinded to group assignments. Group assignments will be disclosed only after the completion of the statistical analysis.

### Interventions

All the groups receive standard treatment, including controlling blood pressure, blood glucose, blood lipid, and symptomatic treatment.

In this trial, 0.25×40 mm needles manufactured by HUATO, Suzhou, China, and the SDZ-III electroacupuncture apparatus by Suzhou Medical Appliance, Jiangsu, China, will be used. Interventions in all groups will be practiced by registered practitioners of TCM with at least 5 years of clinical experience in acupuncture. Acupuncturists will be trained in the study protocol and standardized acupuncture manipulations before study initiation.

### Electroacupuncture Group

Participants in the electroacupuncture group will receive electroacupuncture at Baihui (GV20), Shenting (GV24), Neiguan (PC6), Zusanli (ST36), Fenglong (ST40), and Taichong (LR3). Acupoints PC6, ST36, ST40, and LR3 will be applied bilaterally. All acupoints will be punctured to standardized needling depths and manipulated through lifting, thrusting, or twisting to induce the Deqi sensation. Three paired alligator clips from the electroacupuncture apparatus will be attached to GV20 and GV24, and to the homolateral ST36 and ST40, with a disperse-dense frequency (2/15 Hz) and a patient-tolerable current intensity. The precise locations and manipulations of these acupoints are depicted in Table 1 and Figure 2. This treatment will be administered over a period of 12 weeks, with sessions occurring once daily, 3 times per week.

**Table 1:** Locations and manipulations of acupoints in the electroacupuncture group.

Acupoints	Location	Manipulation
Baihui (GV20)	On the midline of the head, 5 cm directly above the midline of the head, approximately on the midpoint of the line connecting the apexes of both ears.	Horizontal insertion with a depth of 1 cm is applied with a small-amplitude, high-frequency twisting technique of the reinforcing method for 1 min.
Shenting (GV24)	On the midline of the head, 0.5 cm directly above the midline of the head.	Horizontal insertion with a depth of 0.5-0.8 cm is applied with a small-amplitude, high-frequency twisting technique of the reinforcing method for 1 min.
Neiguan (PC6)	On the forearm, 2 cm above the transverse crease of the wrist, between the tendons of musculus palmaris longus and musculus flexor radialis.	Vertical insertion with a depth of 1.0-1.5 cm is applied with the twisting, lifting, and thrusting technique of the reducing method for 1 min.
Zusanli (ST36)	On the outer side of the calf, 3 cm directly below Dubi, and 1 finger-breadth lateral to the anterior border of the tibia.	Vertical insertion with a depth of 0.7-1.0 cm is applied with the twisting of 360°, with a frequency of 120-160 revolutions per minute of the mild reinforcing-reducing method for 1 min.
Fenglong (ST40)	One finger-breadth lateral to Tiaokou and at the midpoint of the line joining Dubi and the tip of the external malleolus.	Vertical insertion with a depth of 1.0-1.5 cm is applied with the twisting, lifting, and thrusting technique of the reducing method for 1 min.
Taichong (LR3)	On the dorsum of the foot, in the depression distal to the junction of the first and second metatarsal bones.	Vertical insertion with a depth of 1.0-1.5 cm is applied with the twisting, lifting, and thrusting technique of the reducing method for 1 min.

### The SEA Group

In the SEA group, 9 nonacupoints will be selected, with their locations detailed in Table 2 and Figure 2. The selection of nonacupoints in the SEA group involves choosing points situated on nonmeridians yet proximal to the acupoints selected in the electroacupuncture group, a common approach in sham acupuncture design [30]. A shallow needling technique (approximately 5 mm depth) will be used at these nonacupoints, eschewing both reinforcing-reducing techniques and the elicitation of the *Deqi* sensation. The SEA device will be connected, with its connecting wires internally disconnected. The number and frequency of treatments will mirror those of the electroacupuncture group.

**Table 2:** Location of nonacupoints in the sham electroacupuncture group.

Nonacupoint	Location
Nonacupoint 1	At the midpoint of the line connecting the Fengchi acupoint (GB20) and the Anmian acupoint (Extra).
Nonacupoint 2	At the midpoint of the line connecting the Shenting acupoint (GV24) and Yintang acupoint (EX-HN 3).
Nonacupoint 3	On the palmar side of the forearm, on a level with Neiguan (PC6) and 1 cm radialis to it (between the Pericardium Meridian of Hand-Jueyin and the Lung Meridian of Hand-Taiyin).
Nonacupoint 4	In the middle of the Yanglingquan acupoint (GB34) and Zusanli acupoint (ST36; between the gallbladder and bladder meridian).
Nonacupoint 5	In the middle of the Fenglong acupoint (ST40) and the Zusanli acupoint (ST36; between the gallbladder and bladder meridian).
Nonacupoint 6	At the midpoint of the dorsum of the foot; the 3rd and 4th metatarsal bones join the anterior depression.

### Waiting List Group

No acupuncture treatment will be given in the waiting list group for the first 12 weeks. Participants will be required to complete the assessments at the corresponding time points. Considering the ethical requirements, compensatory treatment will be given after 12 weeks of following up.

The specific flowchart is shown in Figure 1.

### Outcomes

#### Baseline Comparability

We first confirmed the comparability of the baseline by independent 2-tailed *t* test for continuous variables and chi-square tests for categorical variables, all *P>*.05.

#### Outcome Measures

In this trial, both primary and secondary outcomes will be assessed at baseline, upon intervention completion (week 12), and during the 12-week follow-up period (week 24).

#### Primary Outcome: Montreal Cognitive Assessment

The Montreal Cognitive Assessment (MoCA) [31] tests key areas such as executive function, verbal fluency, orientation, calculation, abstract thinking, delayed recall, visual perception, naming, attention, and concentration. A systematic review [32] indicates that the MoCA effectively detects MCI. Furthermore, the MoCA is a straightforward, independent cognitive screening tool with superior sensitivity (100%). Its memory test encompasses more words, fewer learning trials, longer recall delays, and a shorter assessment duration, rendering it appropriate for clinical applications. It demonstrates excellent test-retest reliability and positive and negative predictive values for MCI and AD [31]. Therefore, we used the MoCA test as our primary outcome measure to ensure maximum diagnostic accuracy.

#### Secondary Outcomes

##### Mini-Mental State Examination

The Mini-Mental State Examination (MMSE) [33] is a comprehensive 30-question assessment tool evaluating cognitive functions, including attention and orientation, memory, registration, recall, computation, language abilities, and the capacity to draw complex polygons [34]. However, an increasing number of studies indicate that the MMSE’s effectiveness in clinically detecting MCI is limited [34-36]. This implies that the MMSE is insufficient for identifying subtle cognitive changes in patients with MCI, particularly in cases of dementia without significant memory decline [34]. Consequently, in this study, the MMSE scale was used as a secondary outcome measure, complementing the MoCA scale to minimize the occurrence of false positives.

##### Blood Pressure Condition

Blood pressure measurements both in the clinic and through ambulatory monitoring were included. Clinic-based blood pressure measurement has primarily relied on the auscultatory method, using mercury, aneroid, or hybrid sphygmomanometers. Patients are advised to abstain from smoking, consuming caffeinated beverages, or exercising for 30 minutes before the measurement of their blood pressure [37]. In the clinic, at least 2 blood pressure readings will be obtained with an interval of 1 to 2 minutes, and the average of these readings will be considered. Should the difference between the first and second blood pressure readings exceed 10 mm Hg, a third measurement is advised, with the average of the latter 2 readings being taken.

The monitor should be fitted only after the patient has been in a relaxed state for at least 5 minutes. Blood pressure is subsequently measured in both arms, and an appropriately sized cuff is placed on the nondominant arm should the difference in systolic blood pressure be less than 10 mm Hg. In addition, an automatic noninvasive portable 24-hour dynamic blood pressure monitor will be attached to the nondominant arm for measuring brachial artery blood pressure. The monitoring frequency typically ranges from every 15 to 30 minutes during the day to every 30 minutes at night. The health care provider will instruct the patient to ensure that the monitor remains connected, to continue performing normal daily activities, and to keep the monitor arm stable and level with the heart throughout the 24 hours. A valid 24-hour ambulatory blood pressure monitoring session necessitates at least 14 readings during the day and at least 7 readings at night [38]. The computer will automatically calculate and record the following parameters: 24-hour mean systolic blood pressure, 24-hour mean diastolic blood pressure, daytime mean systolic blood pressure, daytime mean diastolic blood pressure, night mean systolic blood pressure, night mean diastolic blood pressure, along with the white coefficient of variance (defined as the ratio of the SD of blood pressure to the average systolic and diastolic blood pressure in the same time period) for daily and nocturnal systolic and diastolic blood pressures.

##### Verbal Fluency Test

Impaired executive function represents a principal manifestation of cognitive impairment related to hypertension. The Verbal Fluency Test demonstrates sensitivity in differentiating various cognitive impairments, with varying scores distinguishing between cognitively normal individuals and those exhibiting patterns of AD or MCI. Both in clinical and experimental contexts, the Verbal Fluency Test is extensively used for the diagnosis and assessment of a variety of neurological disorders [39].

##### The Shape Trail Test

The Shape Trail Test (STT) is a variant of the original Trail Making Test, known for its sensitivity in evaluating visual search and sequencing abilities, and is applied in cognitive function assessments. This study predominantly uses the STT, comprising 2 components, A and B. The STT-A assesses language and attention capabilities, whereas the STT-B focuses more extensively on executive function and memory [40].

##### Auditory Verbal Learning Test

The Verbal Learning Test is considered the most reliable predictor of the transition from MCI to AD. In this study, the primary tool used is the Auditory Verbal Learning Test–Huashan version. This version uses the principles and methods of the California Verbal Learning Test, a standardized Verbal Learning Test, and comprises both short-term and long-term delayed recall, making it apt for detecting cognitive impairment in patients with impaired memory [41].

##### Activity of Daily Living

Activities of Daily Living (ADL) are an effective instrument for dementia screening and are well-suited for assessing the daily functional abilities of patients with MCI [42]. This study predominantly uses ADL to diagnose hypertension-related cognitive impairment, encompassing both basic ADL and instrumental ADL. The former pertains to the fundamental skills necessary for independent living, while the latter involves capabilities for more complex daily and social activities [43].

##### Hamilton Anxiety Scale and Hamilton Depression Scale

This scale also serves to assess the anxiety or depression status of the participants over the past week, using a combination of conversation and observation. Research has indicated that cognitive impairment is bidirectionally associated with higher anxiety and depression scores. In this study, the Hamilton Anxiety Rating Scale and the Hamilton Depression Rating Scale will be used to assess hypertension combined with cognitive impairment [44].

##### Pittsburgh Sleep Quality Index

Sleep disorders are known to contribute to various metabolic disorders, including obesity, diabetes, and dyslipidemia, all of which are key factors in high blood pressure [45]. The Pittsburgh Sleep Quality Index evaluated sleep quality through 7 aspects: sleep quality, time to fall asleep, sleep duration, sleep efficiency, sleep disturbance, use of hypnotic drugs, and daytime dysfunction [46].

##### Transcranial Doppler

Transcranial Doppler (TCD) is a useful tool in the diagnosis and treatment of clinical cerebrovascular diseases. In particular, TCD can be used to assess the effects of cerebral artery stenosis and occlusion on cerebral hemodynamics [47]. Hypertension is the major vascular risk factor for cerebral small vessel disease. TCD is a noninvasive method that allows the monitoring of microvascular hemodynamic functional integrity to help guide therapies aimed at the cerebral microcirculation and neurovascular unit [48]. At the same time, studies have shown that cerebral hypoperfusion is associated with both cognitive decline and white matter lesions. Therefore, TCD can explore the cognitive impairment in the midbrain hemodynamic and small vessel disease caused by the relationship between the brain lesions [49]. Important parameters measured in this study include the mean velocity, resistance index, and pulse index within both cerebral arteries.

##### Microcirculation-Related Palliation

The microcirculation, often regarded as a hidden organ, consists of the smallest blood vessels, including resistance arterioles, capillaries, and venules [50]. The endothelium mediates the vasomotor tone of the microcirculation through the release of vasodilators (such as nitric oxide [NO]) and vasoconstrictors (such as endothelin-1 [ET-1]). Concurrently, impaired vasodilation, mediated by reduced NO production, is a characteristic feature of endothelial dysfunction [51]. Consequently, NO and ET-1 will be used as biomarkers to assess microcirculation indicators in patients.

### Safety Evaluation

Adverse reactions, including needle-sickness, needle stagnation, needle bending, broken needle, and subcutaneous hematoma, will be closely monitored during the treatment process. Concurrently, a detailed analysis will be conducted on the correlation between all adverse events and acupuncture to rigorously evaluate the safety of acupuncture treatment. All adverse events will receive appropriate intervention, and serious adverse events will be reported to the ethics committee immediately.

### Data Management and Quality Control

Quality control will be implemented for all records of clinical trials, including case reports, needling operation lists, blood pressure monitoring forms, cognitive assessment scales, among others, with strict control of the time window for follow-up collection to ensure that the data are accurate, complete, truthful, and timely. These records shall not be omitted or arbitrarily altered.

A specialized quality control team will be established to conduct regular on-site inspections. Simultaneously, a comprehensive quality control system will be established during the implementation of the project, and we will formulate a series of standard operating procedures, including standard operating procedures for the study process, the acupuncture treatment, and the follow-up assessment. Furthermore, the Data Inspection Committee has been commissioned to regularly review and assess the data accumulated from the clinical trials, thereby evaluating the quality, efficacy, and safety of the trials.

### Sample Size

According to the literature [52], acupuncture treatment increased the mean MoCA score by 2.83 (SD 1.13); the mean change in MoCA in the sham acupuncture group was 1.97 (1.38), waiting group, the mean change in MoCA was 0.48 (SD 1.65); the test level was α=0.01; power was 95%; and it was calculated by the PASS (version 15.0; NCSS, LLC) software to obtain a sample size of 66, considering a 20% dropout rate, 84 cases were proposed to be included in each group, and 252 participants were finally included.

### Statistical Analysis

Statistical analysis was performed using SPSS (version 27.0) software. All patients who completed the randomization and received the overall course of treatment were included in the efficacy analysis to ensure the integrity and comparability of the data. For different types of data, the following statistical methods will be used for analysis:

1. Data types and test methods: ×2 test is used for count data, and Shapiro-Wilk and Levene test are used for measurement data to analyze the independence, normality, and homogeneity of variance of data.
2. Comparison between groups: for normal distribution and homogeneity of variance, 1-way ANOVA with completely random design was used for comparison between groups. If the variance is not uniform, the Kruskal-Wallis rank sum test of the single-factor nonparametric method of completely random design is used for comparison between groups. For the measurement data conforming to the skewed distribution, the Kruskal-Wallis rank sum test of the single-factor nonparametric method of completely random design was used for comparison between groups.
3. Hypothesis testing and significance level: The count data are expressed as a ratio (%) using the chi-square test or Fisher exact test. The 2-sided test method was used uniformly, and the test statistics and corresponding *P* values were recorded. *P*<.05 was used as a criterion for judging significant statistical significance.

## Results

The study protocol has been approved by the institutional review board of the First Affiliated Hospital of Tianjin University of TCM. This study was registered on April 26, 2024, with the Chinese Clinical Trial Registry. Data collection began in May 2024 and ended in April 2025. Currently, data from this trial are in the collection phase, and no data analysis has been performed. As of January 1, 2025, we have collected data from 65 patients. The results of this trial are expected to be submitted for publication in July 2026.

## Discussion

### Overview

Hypertension is the leading risk factor for the global disease burden, a major cause of cardiovascular and cerebrovascular morbidity and mortality in China, and one of the most prevalent chronic diseases [53]. Microcirculation plays an important role in maintaining peripheral vascular resistance and blood pressure stability. Abnormal microvascular blood perfusion is an early manifestation of hypertension [54]. At the same time, cerebral blood flow regulation disorder and cerebral hypoperfusion have direct influence on cognitive function [13]. Therefore, MD is the common pathophysiological basis of hypertension and MCI.

Clinical findings suggest that TCD markers of cerebral flow regulation in patients with hypertension are strongly associated with cognitive ability, with poorer cognitive function associated with enhanced dynamic brain autoregulation in the middle cerebral artery [55]; The content of NO is closely related to oxidative stress response, which can activate the nuclear factor kappa-light-chain-enhancer of Activated B Cells pathway, and then cause inflammation in vivo, promote the release of proinflammatory mediators in the blood vessel wall, and accelerate the occurrence and development of hypertension and vascular complications; ET-1, as an important substance regulating vasoconstriction, can promote vascular smooth muscle cell proliferation, fibrosis, and inflammation, and has important significance in the pathophysiology of vascular endothelium [56]. All the above can further affect the microcirculation index by affecting the vascular endothelium.

Acupuncture, as a special treatment method of TCM, can regulate blood pressure by regulating the neuroendocrine system, improving metabolic activities, changing gene expression related to blood pressure, and so on [57-59]. At the same time, it has been proved that acupuncture can restore the decreased cognitive function to some extent by regulating the hypothalamic oxidative stress pathway, proinflammatory cytokine [60] and improving the brain white matter injury [61].

According to previous studies, acupuncture stimulation of LR3 and ST36 with electroacupuncture and manual acupuncture can reduce the symptoms of hypertension and subsequent cognitive dysfunction in spontaneously hypertensive rats60. The acupuncture of DU20 and ST36 can reduce blood pressure, increase microvascular dilation, reduce nerve damage, and restore cognitive dysfunction to a certain extent [62]. In addition, electroacupuncture stimulation of bilateral PC6 can affect the inflammatory pathway mediated by the regulation of AngII-TGF-β and then achieve the effect of antihypertension and improvement of spontaneously hypertensive rat cardiac fibrosis [63]. On the basis of the research results mentioned earlier, we selected GV20, GV24, PC6, ST36, ST40, and LR3 for combination therapy.

In this study, the mechanism of microcirculation is combined with modern medicine to monitor cerebral microvascular hemodynamics by TCD. Endothelium dysfunction biomarkers ET-1 and NO were used to determine the contraction and dilation of cerebral microvessels. The relationship between microcirculation and hypertension and MCI was investigated by combining clinical and experimental methods. In summary, we propose a randomized controlled trial to verify whether the improvement of electrotherapy in HTMCI is related to the regulation of microcirculation indicators by acupuncture, and to provide more clinical evidence of the effectiveness of acupuncture in reducing blood pressure and improving cognitive impairment.

### Limitations

This study aims to provide high-quality evidence on the efficacy and safety of electroacupuncture in patients with HTMCI. However, this protocol design also has limitations. First, it is impossible to accurately judge the causality between hypertension and cognitive impairment; thus, the reverse causality bias of MCI affecting blood pressure may occur. Second, the potential for spontaneous recovery early in cases of hypertension complicated with MCI may have confounding effects on this trial, though the waiting list group could mitigate these impacts.

### Conclusions

This clinical trial aims to compare the efficacy of electroacupuncture versus SEA or waiting list control in the treatment of hypertension with cognitive impairment and to explore its impact on microcirculation through hemodynamic and microcirculatory indices. The results of this trial will contribute to clarifying the microcirculatory mechanisms of electroacupuncture in the treatment of hypertension with cognitive impairment, providing a solid foundation for further research on electroacupuncture therapy.

## Abbreviations

AD: Alzheimer disease
ADL: Activity of Daily Living
ET-1: endothelin-1
HTMCI: hypertension with mild cognitive impairment
MCI: mild cognitive impairment
MD: microcirculation disturbance
MMSE: Mini-Mental State Examination
MoCA: Montreal Cognitive Assessment
NO: nitric oxide
SEA: sham electroacupuncture
STT: Shape Trail Test
TCD: Transcranial Doppler
TCM: traditional Chinese medicine
